# Evaluating the association between gestational diabetes and neonatal hypoglycemia in Taiwan: a retrospective study of 2,149 pregnancies

**DOI:** 10.3389/fendo.2025.1634074

**Published:** 2025-09-04

**Authors:** Shiang-Hua Chang, Pei-Hsiu Hsin, Jia-Juen Lin, Yi-Sun Yang, Shih-Chang Lo, Chien-Ning Huang, Yu-Hsun Wang, Edy Kornelius

**Affiliations:** ^1^ School of Medicine, Chung Shan Medical University, Taichung, Taiwan; ^2^ Department of Internal Medicine, Division of Endocrinology and Metabolism, Chung Shan Medical University Hospital, Taichung, Taiwan; ^3^ Institute of Medicine, Chung Shan Medical University, Taichung, Taiwan; ^4^ Department of Medical Research, Chung Shan Medical University Hospital, Taichung, Taiwan

**Keywords:** gestational diabetes mellitus, neonatal hypoglycemia, neonatal outcomes, pregnancy, retrospective cohort, Taiwan

## Abstract

**Objective:**

To determine whether gestational diabetes mellitus (GDM) is associated with an increased risk of neonatal hypoglycemia and adverse neonatal outcomes in a Taiwanese population.

**Methods:**

We performed a retrospective cohort study of 2,149 women who delivered at Chung Shan Medical University Hospital from 2019 to 2023. GDM was diagnosed by one-step 75-g oral glucose tolerance test (OGTT). Neonatal hypoglycemia was defined as blood glucose <45 mg/dL. Logistic regression was used to estimate odds ratios (ORs) for neonatal outcomes associated with GDM, adjusting for maternal age, body mass index (BMI), and parity. Other neonatal outcomes included preterm birth, low Apgar scores (≤7 at 1 or 5 minutes), neonatal intensive care unit (NICU) admission, or neonatal jaundice.

**Results:**

Of 2,149 pregnancies, 591 (27.5%) were diagnosed with GDM. Neonatal hypoglycemia occurred in 176 newborns (8.2%). The incidence of hypoglycemia was slightly lower in infants of GDM mothers (6.8%) compared to those of non-GDM mothers (8.7%), but this difference was not statistically significant (adjusted OR 0.70, 95% CI 0.48–1.02). GDM was also not significantly associated with other neonatal outcomes, including preterm birth, low Apgar scores, NICU admission, or neonatal jaundice, after adjusting for confounders.

**Conclusions:**

In this Taiwanese cohort with universal GDM screening and management, GDM was not linked to a higher risk of neonatal hypoglycemia or other immediate neonatal complications. These findings suggest that effective prenatal care and glycemic control may mitigate the neonatal risks traditionally associated with GDM, underscoring the importance of management and population-specific factors in outcomes.

## 1 Introduction

Neonatal hypoglycemia is one of the most common metabolic abnormalities in newborns (1, 2)​. It can lead to serious consequences, including seizures and long-term neurodevelopmental impairment, if not recognized and treated promptly (3)​. Infants of diabetic mothers are known to be at particular risk due to fetal hyperinsulinemia induced by maternal hyperglycemia​ (4, 5). Gestational diabetes mellitus (GDM), characterized by glucose intolerance first identified during pregnancy, affects approximately 14% of pregnancies worldwide (6)​. Uncontrolled maternal hyperglycemia in GDM can result in excess fetal insulin production, predisposing the neonate to hypoglycemia after birth​ (7, 8). Beyond hypoglycemia, GDM is associated with other adverse perinatal outcomes such as macrosomia, birth injuries, and respiratory distress (7, 9, 10)​. However, most evidence for these associations comes from Western populations, and outcomes can vary with the quality of GDM management and ethnic background.

Asian populations have a higher prevalence and incidence of GDM, with Chinese women showing rates of 7.9% compared with 4.2% in non-Hispanic Whites (11). In 2019, the incidence among non-Hispanic Asians reached 102.7 per 1,000 live births (RR 1.78), and 90.5 per 1,000 among Chinese women (RR 1.57) (12). However, there remains a relative paucity of data on neonatal outcomes in these groups (13, 14). Prior studies suggest that ethnicity may modulate risk, for instance, a recent analysis in a multi-ethnic cohort found that neonates of Asian mothers had significantly lower odds of developing hypoglycemia compared to those of other ethnicities (OR ~0.54)​ (15). Such findings raise the question of whether the impact of GDM on neonatal hypoglycemia might be less pronounced in Asian settings due to genetic, behavioral, or healthcare factors. To date, few studies in Taiwan or similar Asian populations have specifically examined the relationship between GDM and neonatal hypoglycemia.

Given this gap, we undertook a retrospective study in a Taiwanese medical center to evaluate the association between maternal GDM and the occurrence of neonatal hypoglycemia, as well as other neonatal outcomes. We hypothesized that infants born to mothers with GDM would have a higher risk of hypoglycemia than those born to non-GDM mothers, consistent with pathophysiological expectations, but we also considered that management of GDM might attenuate this risk. Our objectives were to quantify the risk of neonatal hypoglycemia associated with GDM and to assess whether GDM is linked to other perinatal outcomes (preterm delivery, birth weight anomalies, Apgar scores, neonatal intensive care unit (NICU) admission), after adjusting for potential confounding factors. This study aims to provide evidence relevant to Asian populations and inform clinical care in the context of universal GDM screening.

## 2 Methods

### 2.1 Study design and setting

We conducted a retrospective cohort study at Chung Shan Medical University Hospital, a tertiary teaching hospital in Taichung, Taiwan. The study period spanned January 2019 through December 2023. We identified all women who delivered at the hospital during this period using the institutional obstetric database. The study was approved by the hospital’s Institutional Review Board, under the approval number CS2 - 25041.

### 2.2 Study population

**Figure 1:**
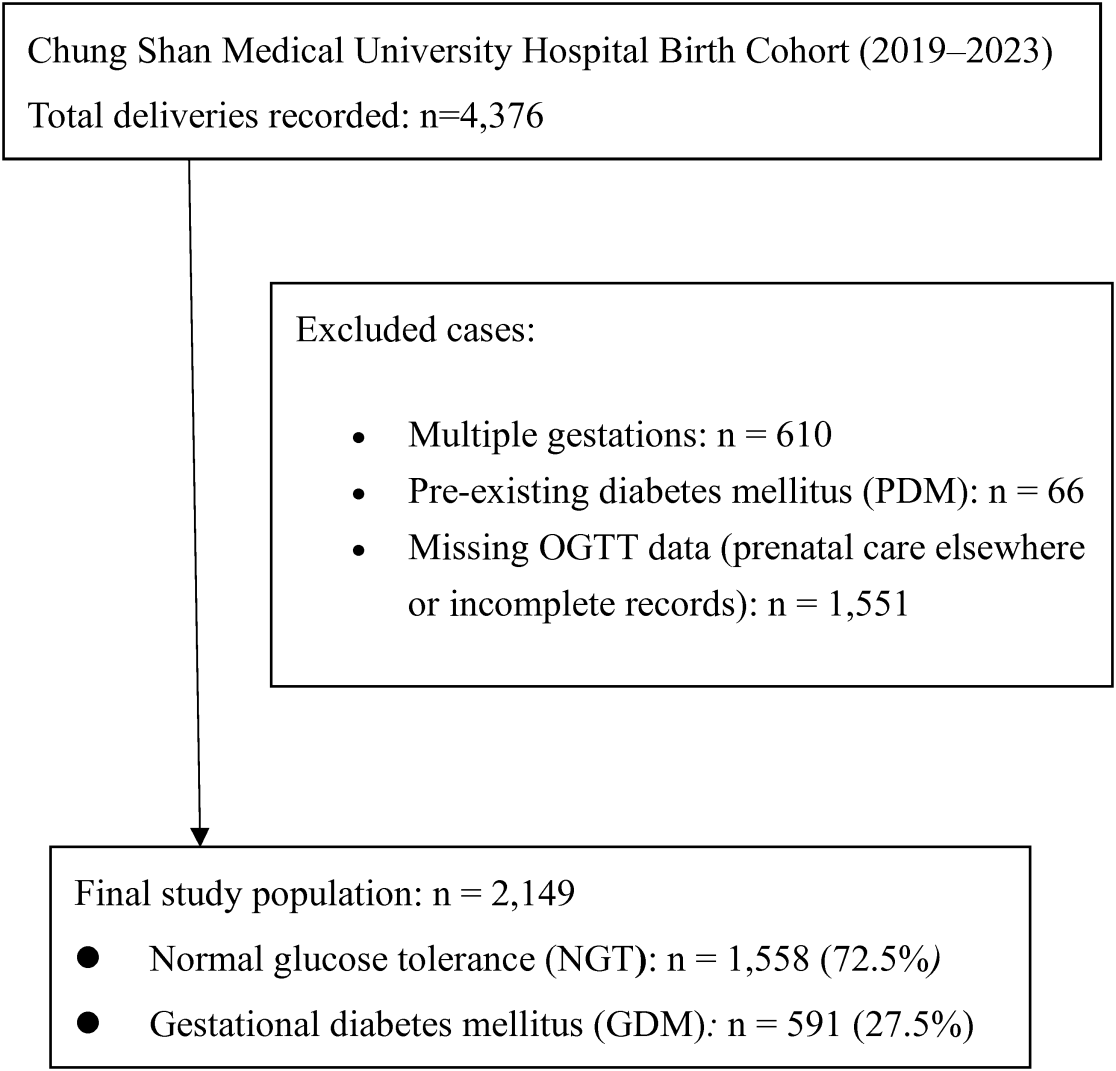
Flow diagram of the study population.

The initial cohort included 4,376 deliveries from 2019 – 2023. We excluded 66 women with pre-existing diabetes mellitus (type 1 or type 2 diabetes diagnosed before pregnancy) to focus on GDM. Women without 75-g oral glucose tolerance test (OGTT) data (n = 1,551) were excluded to maintain consistency in GDM classification and ensure accuracy in exposure measurement. Many of these cases involved patients who received prenatal care at external clinics where OGTT screening data were unavailable in our medical records. After eliminating duplicate or repeat records and restricting to singleton pregnancies, a total of 2,149 women were eligible for analysis (**Figure 1**). Among these, 591 met the criteria for GDM and 1,558 had normal glucose tolerance (NGT) during pregnancy (**Figure 1**).

### 2.3 Exposure - GDM diagnosis

The exposure of interest was GDM, diagnosed according to the International Association of Diabetes and Pregnancy Study Groups (IADPSG) 2010 criteria (16)​. All pregnant women in our hospital received a 75-g OGTT at 24 – 28 weeks’ gestation as part of routine prenatal care. Venous plasma glucose was measured fasting and at 1 hour and 2 hours post-glucose load. GDM was defined as any one or more values meeting or exceeding the IADPSG threshold: fasting ≥92 mg/dL, 1-hour ≥180 mg/dL, or 2-hour ≥153 mg/dL. These criteria align with those recommended by the American Diabetes Association and were universally implemented at our institution during the study period. Women diagnosed with GDM received standard care including dietary counseling, glucose monitoring, and insulin therapy if needed, aiming to maintain euglycemia throughout gestation.

### 2.4 Outcome measures

The primary outcome was neonatal hypoglycemia, defined as a blood glucose concentration <45 mg/dL in the newborn. Per hospital protocol, capillary blood glucose of neonates born to GDM mothers was routinely measured shortly after birth and monitored for the first 24 hours. For this study, we captured any instance of glucose <45 mg/dL documented in the neonate’s chart. Secondary neonatal outcomes included birth weight and gestational age at delivery, Apgar scores at 1 and 5 minutes, incidence of preterm birth (<37 weeks gestation), macrosomia, neonatal jaundice requiring phototherapy, and NICU admission. A low Apgar score was defined as ≤7 at either 1 minute or 5 minutes after birth. Macrosomia was defined as birth weight ≥4,000 g. We also recorded mode of delivery (cesarean *vs*. vaginal) and any stillbirths. All outcome data were obtained from the electronic medical record and newborn nursery logs.

### 2.5 Data collection and variables

Maternal characteristics collected included age at delivery, height, weight, and parity. Pre-pregnancy weight was not uniformly available, so we used the earliest recorded weight in pregnancy (often at the first prenatal visit) to calculate body mass index (BMI) (kg/m²) with the recorded height. We also extracted each woman’s OGTT glucose values (fasting, 1-h, 2-h). Infant sex and birth outcomes were recorded from delivery records. The data were compiled and checked for completeness; any implausible or missing values were verified against source charts when possible.

### 2.6 Statistical analysis

We first compared maternal and infant characteristics between the GDM and NGT groups. Continuous variables (e.g., maternal age, BMI, birth weight) were summarized as mean ± standard deviation for approximately normally distributed variables and compared using Student’s t-test, whereas variables with skewed distributions were summarized as median with interquartile range and compared using the Wilcoxon rank-sum test. Categorical variables (parity, sex, incidence of outcomes) were compared using Chi-square test when the expected frequency in all cells was ≥5, and Fisher’s exact test was applied otherwise. These results are presented in **Table 1**.

**Table 1:** Baseline characteristics of patient with and without gestational diabetes mellitus.

	Total number (N = 2149)	NGT (N = 1558)	GDM (N = 591)	P value
Maternal characteristics				
Age (years)	36.2 (5.0)	35.7 (4.9)	37.7 (4.9)	0.000^***^
Height (cm)	159.9 (5.7)	160.1 (5.9)	159.3 (5.1)	0.008^**^
Weight (kg)	68.9 (10.9)	68.1 (10.2)	71.1 (12.2)	0.000^***^
Body mass index	27.0 (4.8)	26.3 (4.8)	28.0 (4.5)	0.000^***^
OGTT 75g-Fasting glucose	85.8 (7.8)	83.5 (4.3)	92.1 (10.9)	0.000^***^
OGTT 75g-1 hour glucose	140.1 (30.7)	129.5 (22.8)	167.9 (31.3)	0.000^***^
OGTT 75g-2 hour glucose	127.2 (26.9)	117.4 (17.9)	153.0 (29.4)	0.000^***^
Gravidity	1.9 (1.2)	1.9 (1.2)	2.0 (1.2)	0.047^*^
Parity	1.4 (0.7)	1.4 (0.7)	1.5 (0.7)	0.003^**^
Cesarean delivery				0.107
No	1353 (63.0)	997 (64.0)	356 (60.2)	
Yes	796 (37.0)	561 (36.0)	235 (39.8)	
Infant characteristics				
Gestational Age (years)	38.1 (1.8)	38.1 (1.9)	38.0 (1.7)	0.112
Preterm delivery (<37weeks)				0.356
No	1937 (90.1)	1410 (90.5)	527 (89.2)	
Yes	212 (9.9)	148 (9.5)	64 (10.8)	
Stillbirth				0.270
No	2140 (99.6)	1553 (99.7)	587 (99.3)	
Yes	9 (0.4)	5 (0.3)	4 (0.7)	
Gender				0.005^**^
Male	1080 (50.3)	813 (52.2)	267 (45.3)	
Female	1067 (49.7)	745 (47.8)	322 (54.7)	
Infant birth weight	2990.0 (2740.0 - 3250.0)	2975.5 (2730.0 - 3220.0)	3050.0 (2750.0 - 3320.0)	0.003^**^
Macrosomia				
No	2138 (99.5)	1553 (99.7)	585 (99.0)	0.044^*^
Yes	11 (0.5)	5 (0.3)	6 (1.0)	
Infant birth height (cm)	49.8 (4.4)	49.8 (4.1)	49.8 (0.2)	0.751
Apgar score-1 minute after birth	8.7 (0.9)	8.7 (0.9)	8.7 (1.1)	0.264
≤7 points	140 (6.5)	96 (6.2)	44 (7.4)	0.282
>7 points	2009 (93.5)	1462 (93.8)	547 (96.6)	
Apgar score-5 minute after birth	9.8 (0.9)	9.8 (0.8)	9.8 (1.0)	0.213
≤7 points	28 (1.3)	18 (1.2)	10 (1.7)	0.327
points	2121 (98.7)	1540 (98.8)	581 (98.3)	
Infant birth blood glucose (mg/dl)	64.7 (37.5)	63.5 (36.6)	68.0 (39.6)	0.175
Neonatal hypoglycemia (<45 mg/dl)				0.139
No	1973 (91.8)	1422 (91.3)	551 (93.2)	
Yes	176 (8.2)	136 (8.7)	40 (6.8)	
Neonatal jaundice				0.897
No	1557 (72.5)	1130 (72.5)	427 (72.3)	
Yes	592 (27.5)	428 (27.5)	164 (27.7)	
NICU admission				0.506
No	1919 (89.3)	1387 (89.0)	532 (90.0)	
Yes	230 (10.7)	171 (11.0)	59 (10.0)	
Neonatal admission days	6.2 (8.1)	6.1 (8.1)	6.2 (8.2)	0.851

^*^P<0.05, ^**^P<0.01, ^***^P<0.001; NGT, normal glucose tolerance; GDM, gestational diabetes mellitus; OGTT, Oral glucose tolerance test.

Data are presented as mean ± standard deviation, median (interquartile range), or number (percentage). Student’s t-test was used for normally distributed continuous variables, Wilcoxon rank-sum test for skewed continuous variables, Chi-square test for categorical variables with expected cell counts ≥5, and Fisher’s exact test otherwise.

To assess the association between GDM and neonatal outcomes, we performed logistic regression analyses. For each outcome (neonatal hypoglycemia and other dichotomous outcomes), we built an unadjusted model with GDM status as the predictor, and a multivariable model adjusting for potential confounders. Based on *a priori* knowledge, we included maternal age, maternal BMI, and parity in adjusted models, as these factors may influence both the likelihood of GDM and neonatal outcomes (for example, older or higher-BMI mothers are more prone to GDM​ and also to certain obstetric risks) (7). Adjusted odds ratios (aOR) with 95% confidence intervals were calculated. We confirmed there were no strong collinearity issues among covariates. Results of these analyses are shown in **Table 2**. A two-tailed p-value <0.05 was considered statistically significant. All statistical analyses were conducted using SPSS Statistics version 26 (IBM Corp., Armonk, NY). This study is reported following the STROBE guidelines for observational studies.

**Table 2:** The risk of neonatal hypoglycemia and various outcomes in patients with GDM.

	NGT (%)	GDM (%)	OR	95%CI	aOR	95%CI
Neonatal hypoglycemia (<45mg/dl)	136(8.7)	40 (6.8)	0.76	(0.53 - 1.10)	0.7	(0.48 - 1.02)
Cesarean delivery	561(36)	235 (39.8)	1.17	(0.97 - 1.43)	0.94	(0.76 - 1.15)
Preterm delivery	148(9.5)	64(10.8)	1.16	(0.85 - 1.58)	1.01	(0.74 - 1.40)
Macrosomia	5(0.3)	6(1)	3.19	(0.97 - 10.48)	2.34	(0.69 - 7.93)
Neonatal jaundice	428(27.5)	164(27.7)	1.01	(0.82 - 1.25)	1.01	(0.82 - 1.26)
Apgar score-1 minute after birth ≤7 point	96(6.2)	44(7.4)	1.23	(0.85 - 1.77)	1.09	(0.74 - 1.59)
Apgar score-5 minute after birth ≤7 point	18(1.2)	10(1.7)	1.47	(0.68 - 3.21)	1.2	(0.54 - 2.68)
NICU admission	171(11)	59(10)	0.9	(0.66 - 1.23)	0.81	(0.59 - 1.12)

Data are presented as number (percentage). Categorical variables were compared using the Chi-square test when the expected frequency in all cells was ≥5, and Fisher’s exact test otherwise.

GDM, Gestational diabetes mellitus; NGT, normal glucose tolerance; NICU, neonatal intensive care unit; OR, odds ratio; aOR, adjusted odds ratio, adjusted for maternal age, parity and body mass index.

## 3 Results

### 3.1 Participant characteristics

A total of 2,149 mother–infant pairs were analyzed, including 591 (27.5%) mothers with GDM and 1,558 (72.5%) with normal glucose tolerance. Maternal and neonatal baseline characteristics by GDM status are summarized in **Table 1**. The mean maternal age was 36.2 years (± 5.0). Women with GDM were significantly older on average than those without GDM (37.7 ± 4.9 *vs*. 35.7 ± 4.9 years, p<0.001). GDM mothers also had a higher mean BMI (28.0 ± 4.5 *vs*. 26.3 ± 4.8, p<0.001) and higher weight at baseline, consistent with known risk factors for GDM. As expected, OGTT glucose levels were markedly higher in the GDM group at all time points (mean 1-h glucose 167.9 *vs*. 129.5 mg/dL in NGT, for example; p<0.001). GDM pregnancies had marginally higher gravidity and parity than NGT pregnancies (median parity 1.5 *vs*. 1.4, p<0.01), suggesting a somewhat greater proportion of multiparas in the GDM group.

Regarding obstetric outcomes, the overall rate of cesarean delivery was 37.0%. GDM was not associated with a significantly higher cesarean rate (39.8% in GDM *vs*. 36.0% in NGT, p=0.107). Mean gestational age at delivery was similar between groups (38.0 weeks for GDM *vs*. 38.1 weeks for NGT, p=0.11), and the incidence of preterm birth (<37 weeks) was comparable (10.8% *vs*. 9.5%, p=0.36). There were 9 stillbirths in the cohort (0.4% overall), with no significant difference by GDM status (0.7% GDM *vs*. 0.3% NGT, p=0.27). Newborn sex distribution differed slightly: the GDM group had a higher proportion of female infants (54.7% *vs*. 47.8%, p=0.005).

Neonatal anthropometrics showed that the median birth weight in the GDM group was 3050 g (IQR 2750 – 3320), compared to 2976 g (2730 – 3220) in the NGT group (p=0.003). Despite this modest shift, the frequency of macrosomia was low in both groups. Only 0.5% of all infants weighed ≥4000 g at birth, with a slightly higher incidence in GDM (1.0% *vs*. 0.3% in NGT; 6 cases *vs*. 5 cases, p=0.044). No significant difference was observed in birth height (median 49.8 cm in both groups). Apgar scores were generally high in both groups. The proportion of infants with a 1-minute Apgar ≤7 was 7.4% in GDM *vs*. 6.2% in NGT (p=0.28), and for 5-minute Apgar ≤7 it was 1.7% *vs*. 1.2% (p=0.33); thus GDM did not appreciably impact immediate neonatal adaptation as measured by Apgars.

### 3.2 Neonatal hypoglycemia and other outcomes

Across the entire cohort, 176 neonates (8.2%) experienced neonatal hypoglycemia. The occurrence of hypoglycemia was actually lower among infants of GDM mothers (40/591, 6.8%) compared to infants of non-GDM mothers (136/1,558, 8.7%), although this difference did not reach statistical significance (p=0.14)​. In unadjusted analysis, GDM mothers had a crude OR of 0.76 (95% CI 0.53 – 1.10) for neonatal hypoglycemia relative to non-GDM mothers​. After adjusting for maternal age, BMI, and parity in a logistic model, the association remained non-significant (adjusted OR 0.70, 95% CI 0.48 – 1.02; p=0.06) (**Table 2**).

The risk of preterm delivery was similar (aOR 1.01, 95% CI 0.74 – 1.40 for GDM *vs* NGT). The likelihood of cesarean delivery, as noted, was not elevated by GDM after controlling for confounders (aOR 0.94, 95% CI 0.76 – 1.15). There was no significant difference in NICU admission rates (10.0% in GDM *vs* 11.0% in NGT; aOR 0.81, 95% CI 0.59 – 1.12)​. Likewise, neonatal jaundice occurred at similar frequencies (27.7% *vs* 27.5%; aOR 1.01, 95% CI 0.82 – 1.26). GDM status did not affect Apgar scores; the adjusted odds of a 1-minute Apgar ≤7 in GDM *vs* non-GDM were 1.09 (0.74 – 1.59), and for 5-minute Apgar ≤7 were 1.20 (0.54 – 2.68), neither significant. We did observe that infants of GDM mothers had slightly higher mean blood glucose at birth (mean ~68 mg/dL *vs* 63.5 mg/dL in NGT, p=0.17), consistent with fewer hypoglycemia cases, although this difference was not significant.

## 4 Discussion

In this large retrospective study of pregnancies in Taiwan, we found that GDM was not associated with a statistically significant increase in neonatal hypoglycemia. In fact, the observed rate of hypoglycemia was slightly lower in the GDM group than in the non-GDM group, although this difference did not reach significance. These results initially seem counterintuitive given the well-established pathophysiology linking maternal hyperglycemia to neonatal hypoglycemia via fetal hyperinsulinemia (3, 7, 8)​. Traditionally, infants of diabetic mothers have been considered at high risk for hypoglycemia, and previous studies in predominantly Western populations have reported substantially elevated risks. For example, a population-based study in Germany (where GDM prevalence was ~5%) found an 11-fold higher odds of neonatal hypoglycemia in infants born to GDM mothers compared to normoglycemic mothers (OR 11.71, 95% CI 7.49 – 18.30) (7)​. Our findings diverge from such reports, suggesting that the relationship between GDM and neonatal outcomes may be more nuanced and influenced by context.

Several factors could explain why GDM did not confer an increased hypoglycemia risk in our cohort. First, the management of GDM in our setting was likely effective in mitigating fetal hyperglycemia. All women diagnosed with GDM received dietary interventions and glucose monitoring, with insulin therapy as needed. The generally low incidence of macrosomia (1% in GDM infants) and the slightly higher mean neonatal glucose levels in the GDM group point toward good glycemic control during pregnancy. Prior research has shown that treatment of even mild GDM can significantly reduce neonatal complications. In randomized trials, intensive management of GDM led to reduced rates of neonatal hypoglycemia and macrosomia (17, 18)​. A recent meta-analysis of 18 trials found that lifestyle interventions (diet and exercise) in GDM pregnancies lowered the risk of neonatal hypoglycemia by about 27% (RR 0.73, 95% CI 0.54 – 0.98)​ (19). It is plausible that the proactive management in our cohort achieved similar benefits, blunting the impact of maternal hyperglycemia on the neonate.

Second, ethnic and physiological differences in our predominantly Asian population may modulate the impact of GDM on the infant. Asian women tend to develop GDM at lower BMIs and may have different patterns of insulin resistance and beta-cell function compared to Western populations (20)​. These differences could result in less fetal overnutrition and hyperinsulinemia for a given degree of maternal glycemia. Notably, Oladimeji et al. recently reported that among GDM pregnancies in New Zealand, neonates of Asian mothers had about half the odds of developing hypoglycemia compared to those of European mothers (15)​. This suggests a potential protective effect or lower susceptibility to hypoglycemia in Asian infants, which could be due to genetic factors or lifestyle factors (such as diet composition) that influence glucose metabolism. In our study, all participants were Taiwanese, so there was no ethnic heterogeneity; however, our findings align with the notion that the risks associated with GDM might be attenuated in an Asian context.

Third, the criteria used to diagnose GDM (IADPSG 2010) identify relatively mild degrees of hyperglycemia. Many women in our GDM group likely had moderate glucose elevations that were well-managed, rather than overtly high blood sugar levels. By expanding the GDM definition to any single abnormal OGTT value, the IADPSG criteria increase GDM prevalence but the average severity of hyperglycemia among GDM cases is lower (16)​. Some of these women might not have been labeled GDM under older criteria. It is possible that in our cohort, a sizable fraction of GDM cases were “mild GDM” that, with proper management, did not translate into neonatal metabolic disturbances. This could partially explain why our GDM group’s neonatal outcomes were comparable to the NGT group. It raises an interesting point for clinicians: with current diagnostic criteria and good treatment protocols, the historical complications of GDM can be markedly reduced.

Our findings must be interpreted in light of comparisons with other studies. While, as noted, several studies indicate GDM increases neonatal hypoglycemia risk (7)​, there is also evidence of variation. A study from Saudi Arabia observed a 13% hypoglycemia incidence in infants of GDM mothers (21). In a Japanese tertiary center, Arimitsu et al. reported a neonatal hypoglycemia incidence of 45% among infants of GDM mothers​, but those mothers had relatively poor glycemic indicators (elevated HbA1c and many required insulin) (22)​. That high figure likely reflects more severe GDM cases; by contrast, the 6.8% incidence in our GDM group is markedly lower, reinforcing the importance of glycemic control. Thus, the spectrum of GDM and its management can lead to very different neonatal outcomes. Our study adds to this body of evidence by showing that in a setting of universal screening and treatment, GDM per se may not drastically elevate neonatal risks.

Beyond hypoglycemia, we found no significant association between GDM and other short-term neonatal outcomes. There was a slight increase in birth weight in the GDM group, but the difference was small (median ~75 g) and the incidence of macrosomia remained very low. This contrasts with some prior findings where GDM is linked to macrosomia and related complications (7)​. The low macrosomia rate again likely reflects effective weight and glucose management during pregnancy. We also did not observe higher rates of preterm birth or NICU admissions attributable to GDM. In fact, the need for NICU care was marginally lower for infants of GDM mothers, though not significantly. This could be due to closer monitoring of GDM pregnancies leading to optimal timing of delivery and immediate neonatal care (e.g., proactive feeding to avoid hypoglycemia). The overall similarity in Apgar scores and newborn well-being measures between GDM and non-GDM groups in our study is an encouraging indication that with current obstetric practices, many GDM-associated risks are reducible.

Key strengths of this study include the relatively large sample size from a single institution and the comprehensive data capture through electronic records. We had access to detailed maternal glucose measurements and neonatal blood glucose values, which allowed accurate classification of exposure and outcome. The use of standardized diagnostic criteria and treatment protocols for GDM at our hospital lends consistency to the management each patient received. Additionally, we adjusted for major confounding variables (maternal age, BMI, parity) that differ between women with and without GDM and could influence neonatal outcomes.

However, several limitations should be noted. First, as a single-center retrospective study, the findings may not be generalizable to all settings. Practice patterns and population characteristics in Taiwan (e.g., high rate of GDM screening and treatment, lower obesity prevalence) may differ from other regions. Second, we did not have data on maternal glycemic control indicators such as HbA1c levels or detailed treatment regimens (diet-controlled *vs*. insulin-treated GDM). Another limitation of our study is the exclusion of 1,551 women due to missing OGTT data (~35% of the total cohort). This was primarily due to some women not undergoing OGTT screening at our institution, particularly those receiving prenatal care at external facilities.

Lastly, our study focused on immediate neonatal outcomes. We did not examine longer-term outcomes such as infant growth, neurodevelopment, or the development of metabolic issues in childhood, which are beyond the scope of this report. Some studies suggest that even transient neonatal hypoglycemia can have developmental implications (23)​. It would be valuable to follow this cohort to see if subtle differences emerge later in infancy or childhood.

The results of this study provide a cautiously optimistic message: with current diagnostic criteria and management strategies, GDM pregnancies can have neonatal outcomes almost as good as non-GDM pregnancies in the short term. Clinicians managing GDM in settings similar to ours (well-resourced environments with early screening and intervention) can expect that diligent control of maternal glucose will largely protect against neonatal hypoglycemia and other acute complications. Our data underscore the importance of standard GDM care, diet therapy, blood glucose monitoring, and timely insulin use if needed, in preventing excessive fetal insulin levels. They also suggest that universal screening and treating mild hyperglycemia (as per IADPSG) is not leading to an epidemic of neonatal hypoglycemia; on the contrary, it may be preventing it.

Our study highlights the need for further research in diverse populations. Multicenter studies in Asia could confirm whether the lack of association between GDM and neonatal hypoglycemia holds true broadly, or identify subgroups where risk is higher. Additionally, exploring the mechanistic basis for ethnic differences in neonatal response to GDM could offer insights. Long-term follow-up of infants born to GDM mothers in this population would be valuable to assess whether being exposed to milder hyperglycemia *in utero* (with good neonatal outcomes) has any later effects on metabolic health or development. Finally, cost-benefit analysis of the current universal screening and management paradigm in terms of neonatal outcomes could inform future guidelines: if well-managed GDM has minimal impact on immediate neonatal health, the focus may shift more toward maternal outcomes and long-term child outcomes when evaluating interventions.

In a cohort of over two thousand Taiwanese pregnancies, we found no significant association between gestational diabetes and neonatal hypoglycemia or other immediate neonatal complications. Infants born to mothers with GDM, under a regimen of early diagnosis and active management, had outcomes comparable to those of non-diabetic mothers in the neonatal period. These findings suggest that the adverse impact of GDM on neonates can be substantially mitigated in practice. Ensuring rigorous GDM screening and care is therefore critical in reducing neonatal morbidity. Our study also underscores the importance of considering population-specific factors; results from Western populations may not directly extrapolate to Asian settings. Continued research and surveillance are needed to refine GDM management protocols, with the ultimate aim of improving both maternal and child health while avoiding unnecessary interventions. Overall, this study contributes evidence that effective management of gestational diabetes is associated with healthy neonatal outcomes, providing reassurance to patients and clinicians and supporting the ongoing efforts in diabetes care during pregnancy.

## Data Availability

This population-based study obtained data from the TrinetX platform (accessible at https://trinetx.com/), for which third-party restrictions apply to the availability of this data. The data were used under license for this study with restrictions that do not allow for data to be redistributed or made publicly available. To gain access to the data, a request can be made to TriNetX (join@trinetx.com), but costs might be incurred, and a data-sharing agreement would be necessary.
